# The effect of overweight on cognition in first episode of psychosis. Interaction with sex

**DOI:** 10.1192/j.eurpsy.2023.1053

**Published:** 2023-07-19

**Authors:** V. Sanchez-Gistau, M. Ilaurador, A. Cabezas, M. Sole, M. Algora

**Affiliations:** 1 Early intervention service; 2 Hospital Universitari Institut pere Mata, reus, Spain

## Abstract

**Introduction:**

Previous studies have shown an association between high Body Mass Index (BMI) and worse cognitive performance in obese patients and the general population. Cognitive deficits and overweight are important challenges in the clinical treatment of psychosis but scarcity of studies have investigated this relationship. Moreover the effect of sex in the psychosis research has been neglected since recent years and there is still a lack of studies taking into account the sex differences

**Objectives:**

To determine the effect of overweight and its interaction with sex on cognitive performance in First Psychotic Episodes (FEP)

**Methods:**

We included 159 FEP out-patients (mean age 23.1 years 33.3% females ) in their first year of antipsychotic treatment attending to the Early Intervention Program of the Institut Pere Mata, in Reus. At study entry participants underwent to a comprehensive clinical, biometric and cognitive assessment by standardized neurocognitive battery (MATRICS Consensus Cognitive Battery; MCCB). T scores were converted to standard equivalents (z-scores) based on data from a healthy control group from the same geographical area.

Overweight was defined as BMI≥ 25 according to WHO standards. Two-way ANCOVAS were performed to determine the interaction effect between overweight and sex on the cognitive tests. Ethical approval was obtained by the Committee for Ethical Clinical and Pharmacological Investigation of the Pere Virgili Research Institute

**Results:**

At study entry 85% of participants were on antipsychotics for less than 6 months with a median dose of 300 (200-450) chlorpromazine equivalents (CPMZ) in mg/day. 37.7 % of participants were overweight without differences between men (41.5%) and women (30.2%) (X2 = 1.47; p = 0.22). There were no clinical and treatment differences between overweight and normal weight participants. With regards sex differences, females presented statistically significant higher scores in Calgary depression scale and lower doses of antipsychotics. No differences were found in cognitive performance regarding weight status but we found a significant interaction between sex and overweight in verbal learning memory ( F=6.09; p=0.01). When controlled for depressive symptoms and CPZM equivalents differences continued to be significant. Overweight females performed worse -1.09 ( SD 1.18) than normal weight females -0.23 (SD 1.10); (t= 2.57;p=0.02) in verbal learning memory whereas that difference was not found among overweight and normal weight males (t= -1.10; p=0.27)

**Conclusions:**

Our results provide evidence for sex-differences on cognitive function depending on weight status. Advances in the study of sex differences in FEP would help to target specific treatment strategies

**Disclosure of Interest:**

None Declared

